# Excess healthcare costs of psychological distress in young women: Evidence from linked national Medicare claims data

**DOI:** 10.1002/hec.4641

**Published:** 2022-12-10

**Authors:** Danusha Jayawardana, Brenda Gannon, Jenny Doust, Gita D. Mishra

**Affiliations:** ^1^ Centre for Health Economics Monash Business School Monash University Caulfield East Victoria Australia; ^2^ School of Economics and Centre for the Business and Economics of Health University of Queensland St Lucia Queensland Australia; ^3^ NHMRC Centre for Research Excellence on Women and Non‐Communicable Diseases (CRE WaND) School of Public Health The University of Queensland Herston Queensland Australia

**Keywords:** Australia, healthcare costs, psychological distress, young women

## Abstract

The prevalence of mental health disorders in young adults is increasing, yet there is limited empirical evidence on its economic consequences. We contribute to the literature by estimating the healthcare costs of psychological distress using panel data of young women (aged 18–23 years with a 5‐year follow‐up) from the Australian Longitudinal Study on Women's Health and linked administrative data from Medicare Australia. Our empirical strategy is based on the classical two‐part model of healthcare costs with individual specific fixed‐effects. We complement our analysis with a test for selection on unobservables to address potential concerns of endogeneity. We find that young women with psychological distress have 15% higher annual healthcare costs (excluding hospital costs) than women with no psychological distress. A large proportion of these costs is driven by the use of antidepressants and the services of psychiatrists and psychologists. We further find that women with psychological distress have higher out‐of‐pocket costs on these mental health related services compared to non‐mental health specific services. Additionally, we show that the effect of psychological distress on healthcare costs is highest during the first 6 months of onset, which gradually decreases afterwards. The findings justify the importance of policy initiatives towards early prevention and treatment of psychological distress, especially among young women.

## 1 INTRODUCTION

Mental health disorders are a global health issue where 1 in 10 individuals are affected by a mental illness, accounting for 792 million people worldwide (Ritchie & Roser, 2018). Among them, depression is one of the most common mental health disorders which has the third highest burden of disease globally (WHO, 2008), as well as in Australia (AIHW, 2014). Although depression can affect individuals during all stages of life, the highest prevalence is seen among young people (Beirão et al., 2020; Productivity Commission, 2020; Schubert et al., 2017), especially among young women, as they are twice as likely as men to develop depression (Hyde et al., 2008; Kessler, 2003; Seedat et al., 2009). Moreover, compared to older adults, the proportion of young people experiencing psychological distress has shown a significant increase over the past decade (Burns et al., 2020; Butterworth et al., 2020; Twenge et al., 2019). Given the ongoing rise of psychological distress in young adults, quantifying the healthcare costs attributable to it is important. On the one hand, it is a starting point to signify the magnitude of the problem in terms of monetary terms (Productivity Commission, 2020), while on the other, it provides insights to estimate and predict the current and future economic burden of severe mental disorders. Further, it also provides evidence for policy makers to assess the economic return associated with interventions for prevention and treatment of mental illness (Lee et al., 2017). However, despite such importance, the healthcare consequences of poor mental health in young adults remain relatively unexplored (Bodden et al., 2018; König et al., 2020).

The literature on the economic costs of mental disorders is vast. For instance, several studies have looked at the economic burden of poor mental health in terms of labor market outcomes (Alexandre & French, 2001; Banerjee et al., 2017; Chatterji et al., 2007, 2011; Frijters et al., 2014; Hamilton et al., 1997; Marcotte & Wilcox‐Gok, 2003; Peng et al., 2016), and educational attainment (Currie & Stabile, 2006; Ding et al., 2009; Eisenberg et al., 2009; Fletcher, 2010; Fletcher & Wolfe, 2008). Yet, the healthcare costs associated with the diagnosis and treatment of mental disorders are the most significant direct economic cost (Trautmann et al., 2016).^1^ A few studies have examined such healthcare costs associated with depression, particularly among young adults, which show that depression is correlated with higher healthcare spending (Bodden et al., 2018; Keenan‐Miller et al., 2007; Lee et al., 2017). Merely examining the association between mental disorders such as depression and medical costs can be problematic due to several reasons. First, there is a possibility of negative selection bias due to the socioeconomic gradient in mental health (Byles et al., 2012; Hammarström et al., 2011; Kosidou et al., 2011). Specifically, individuals with mental illness are more likely to be from socioeconomically disadvantaged backgrounds with lower utilization of healthcare services, resulting in an underestimate of healthcare costs. Second, unobserved heterogeneity such as genetic disposition, level of health literacy, attitudes and preferences can be correlated with both mental health and healthcare use, leading to biased estimates (Roy & Schurer, 2013). Third, due to financial concerns, higher medical spending may in turn affect the mental health status of the individual, resulting in reverse causality or simultaneity bias (Hsieh & Qin, 2018).

The prime aim of this study is to address these potential biases to quantify the effect of psychological distress in young women on publicly funded healthcare costs. In doing so, it contributes to the literature on healthcare consequences of mental health disorders in the following ways. First, to our knowledge, this study provides new evidence on the excess healthcare costs attributable to psychological distress in young women. We leverage a nationally representative panel of survey data linked to complete administrative records on healthcare costs from Australia's public health insurance system (Medicare). A key strength of this setting is that it minimizes bias due to misreporting of healthcare utilization and costs (Black et al., 2018; Lebenbaum et al., 2021), allowing us to derive precise and reliable estimates of excess healthcare costs of psychological distress.

Second, we rely on a variety of econometric techniques to account for important sources of endogeneity bias. Our preferred empirical strategy is based on the classical two‐part model of healthcare costs with individual fixed‐effects to capture unobserved individual heterogeneity. We further complement our analysis by applying Oster (2019) method for selection on unobservables to address concerns of potential omitted variable bias. In addition, we also conduct several checks to examine the robustness of our estimates to sample attrition bias and measurement error.

Third, in contrast to previous studies, we examine heterogeneous effects on several dimensions. The type of medical treatment differs with both the severity and chronicity of psychological distress (Druss & Rosenheck, 1999; Tomonaga et al., 2013). Therefore, we first determine the healthcare costs attributable to different severity levels of psychological distress categorized as mild, moderate and severe. Next, we consider the varying effects of chronicity of psychological distress on healthcare costs. Following previous literature, we define chronicity as one‐time onset (reporting of symptoms only in one wave) and consistent onset (reporting of symptoms throughout the sampling frame) (Jones et al., 2018; Pan et al., 2020). Besides, we also conduct a separate analysis on monthly healthcare costs. Importantly, this allows us to provide new insights on the short and long term variations in healthcare costs following the occurrence of psychological distress.

Our findings indicate that a one‐unit increase in K10 score (which represents an increase in the level of psychological distress) increases the conditional costs by 1.6%. Moreover, women with psychological distress (i.e., K10 score >24) have 15% higher annual healthcare costs, than women with no psychological distress. The additional annual cost of having moderate and severe psychological distress among women aged 18–27 years is about $43 million and $96 million, respectively. Additionally, we find that the effect of psychological distress on healthcare costs is highest during the first 6 months of onset, and gradually decreases afterwards. These findings are robust to several sensitivity checks. We support our findings by exploiting the richness of the administrative data on Medicare records. We show that the excess costs of psychological distress are driven by the use of mental health related services such as the use of psychologists, psychiatrists and prescribed antidepressants. We further show that women with psychological distress incur higher out‐of‐pocket costs on these mental health related services compared to non‐mental health specific services.

Policy‐wise, the findings have significant implications. First, the excess healthcare cost of psychological distress is substantial. Second, individuals with psychological distress are more likely to develop major mental health disorders, which further aggravate the burden on healthcare resources. Therefore, our findings underscore the importance of policy interventions for early prevention and treatment of psychological distress to minimize not only the economic consequences of psychological distress, but also the larger costs of other mental illnesses.

The remainder of the paper is organized as follows. In Section 2 we describe the data and the analysis sample. Section 3 outlines the methodology. The empirical results are presented in Section 4, followed by robustness checks in Section 5. Section 6 discusses the findings, and Section 7 concludes.

## 2 DATA

### 2.1 Data and sample

Our data are from the Australian Longitudinal Study on Women's Health (ALSWH), which is an ongoing panel survey. This study uses data from the cohort born in 1989–95 of ALSWH which began in 2013 and was primarily recruited via the Internet and social media platforms (see Mishra et al. (2014) for detailed information on the study design and sample.) To date there are six waves of data collected annually and our analysis is based on an unbalanced panel of women observed from wave 1 in 2013 (aged 18–23) to wave 5 in 2017 (aged 22–27 years). After dropping respondents with missing data on variables of interest, our sample consists of 16,892 women with 53,449 person‐year observations.^2^

### 2.2 Measure of psychological distress

We use the Kessler Psychological Distress Scale (K10) to construct our measure of psychological distress. The K10 scale is an internationally validated screening instrument of psychological distress, based on 10 questions (Kessler et al., 2002, 2003). As a self‐reported measure, it reports how often the respondent experienced psychological distress (such as feelings of worry, anxiety, hopelessness and unhappiness) during the past 4 weeks.^3^ All questions include five response categories from 1 to 5 (1 = none of the time; 2 = a little of the time; 3 = some of the time; 4 = most of the time and 5 = all of the time). The K10 score is calculated by obtaining the sum of the 10 responses. This ranges from 10 (no psychological distress) to 50 (severe psychological distress), meaning a higher score reflects a higher level of psychological distress. In general, a score greater than 24 is considered as an indication of a high level of psychological distress (Cornelius et al., 2013). In our analysis we further adopt the following cut‐off scores to categorize women into four levels; no psychological distress (10–19); mild psychological distress (20–24); moderate psychological distress (25–29) and severe psychological distress (30–50) (Yiengprugsawan et al., 2014).

In addition to the K10 score, there are two measures of depression self‐reported in all waves, except wave 4 (2016). First, a binary indicator refers to whether the individual has been ever diagnosed with or treated for depression. Second, a multi‐item indicator where the individual reports how often they had depression in the last 12 months; (1) never; (2) rarely; (3) sometimes and (4) often. Since K10 assesses mental health symptoms related to depression and anxiety (Kessler et al., 2002), we use both these alternate measures as robustness checks (see Section 5).

**FIGURE 1:**
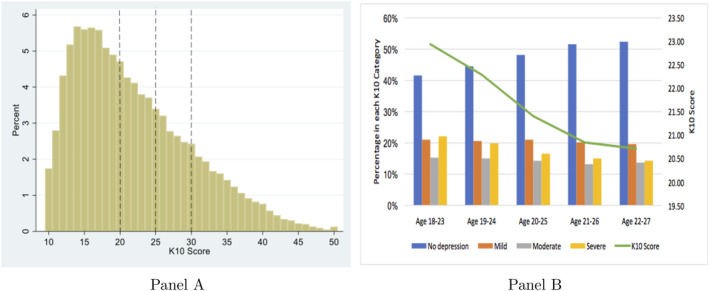
Distribution and dynamics in K10 score. Authors' illustration based on data from the cohort born in 1989–95 of the ASLWH survey. The dotted lines present the cut‐off points for the levels of psychological distress; no psychological distress (<20), mild psychological distress (20–24), moderate psychological distress (25–29) and severe psychological distress (≥30).

Figure 1 illustrates the distribution and dynamics of the K10 score for the pooled sample of women (age 18–27). Almost half of the women (47%) have a score of less than 20 indicating no significant feelings of distress. The percentage of women with mild, moderate and severe psychological distress are 21%, 14% and 18% respectively (Panel A). Panel B shows the dynamics in K10 score by age. K10 score shows a gradual decrease as women get older. The K10 score peaks at a score of 23 when the women are younger (18–23 years) and reduces to a score of 21 after 5 years (22–27 years). Similarly, the percentage of women with no psychological distress increases from 42 to 52 over the 5 years while the percentage of women with severe psychological distress decreases from 22 to 14.^4^

### 2.3 Measure of healthcare costs

Data on healthcare costs are extracted from the linked administrative records of Medicare Australia. Medicare is a universal public health insurance scheme that provides free or subsided healthcare access to all individuals who reside permanently in Australia. The Medicare records include complete data on medical services, which are eligible for rebate through the Medicare Benefits Schedule (MBS) and prescription medications subsidized through the Pharmaceutical Benefits Scheme (PBS).

Several broad categories of medical services are covered under the MBS which includes consultations by general practitioners (GPs), practice nurses, specialists, allied health professionals and other outpatient services such as pathology, diagnostic and imaging, optometry, among many others. However, allied health and specialist attendances are the key categories that include a majority of the mental health related services. Particularly, specialist attendances capture items corresponding to consultations by psychiatrists, and allied health includes psychological services provided by clinical psychologists, psychologists, occupational therapists and social workers, introduced under the Better Access to Mental Health Care initiative in 2006.^5^

The MBS also covers hospital services provided only to private patients. Since it does not capture inpatient services for public patients, we exclude all MBS records of hospital services to maintain consistency. Therefore, our measure of total healthcare cost (or Medicare cost) is the summation of annual costs incurred by each woman for non‐hospital medical services under MBS and pharmaceuticals listed under PBS.^6^ All costs are in 2019 Australian dollars (AUD) adjusted for inflation.

**FIGURE 2:**
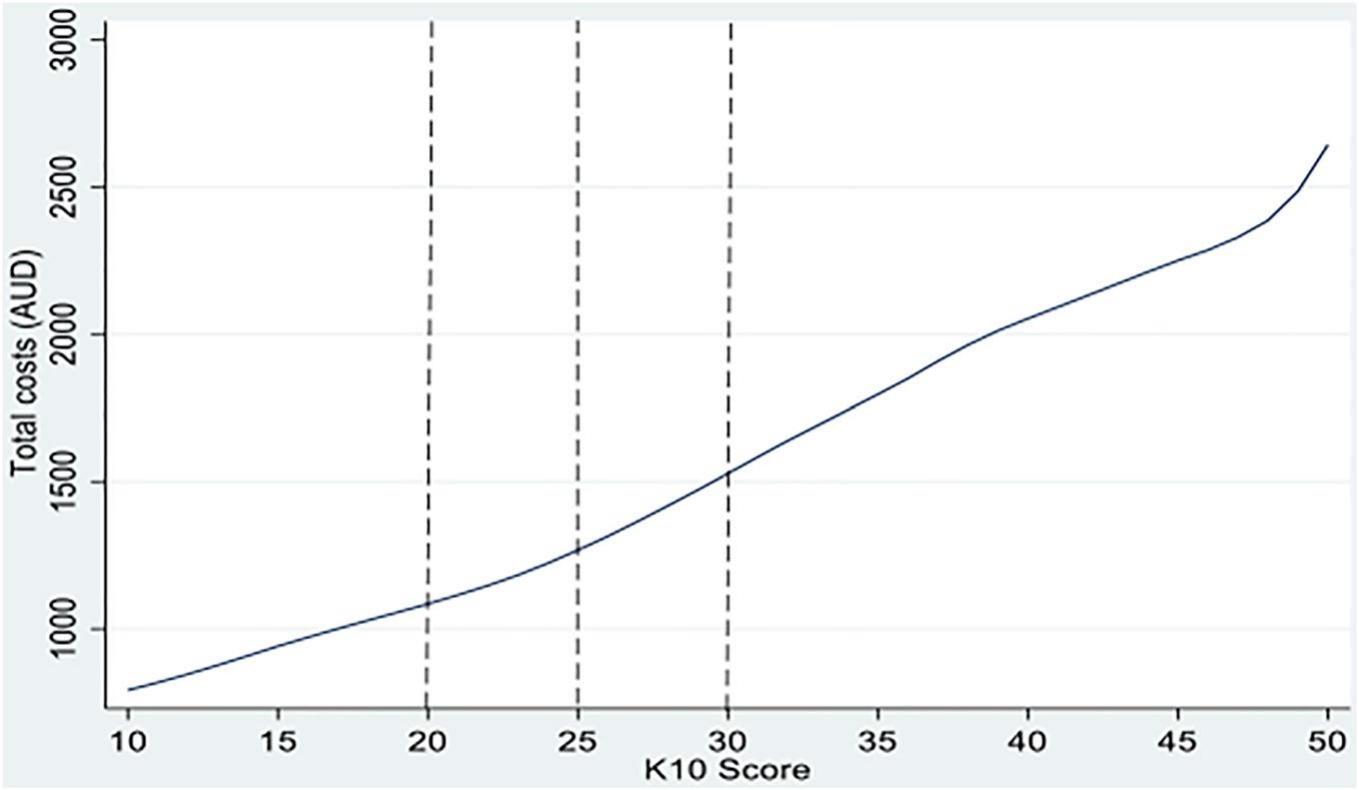
Non‐parametric regression of healthcare costs on K10 score. Data is for the pooled sample (age 18–27), *N* = 53,449. Total costs includes both MBS and PBS costs, and are in 2019 Australian dollars. The dotted lines present the cut‐off points for the levels of psychological distress; no psychological distress (<20), mild psychological distress (20–24), moderate psychological distress (25–29) and severe psychological distress (≥30).

Figure 2 shows the non‐parametric estimates of the association between K10 score and total healthcare costs. The figure depicts a somewhat linear relationship between K10 score and costs, where the costs increase gradually with an increase in K10 score. Compared to the range of no psychological distress, the variation in costs becomes steeper with each level of symptoms. This implies that the effect of psychological distress on healthcare costs is progressive with each level.

Table 1 shows the distribution of healthcare costs by levels of psychological distress for the pooled sample. The mean annual total healthcare cost in the full sample is $1198, and it varies with the level of psychological distress categories (Panel A). Women with severe psychological distress incur $1802 per year as Medicare costs, which is almost double the costs of women with no psychological distress. Women with moderate and mild psychological distress incur an additional $409 (43%) and $187 (20%) than women with no psychological distress respectively. A similar pattern is observed for total MBS (medical services) costs. However, for specific MBS items, mean costs for specialist and allied attendances are considerably greater for women in moderate and severe psychological distress. Specifically, women with severe psychological distress incur three to five times higher costs for total specialist and allied items, compared to women with no psychological distress. This may be due to the utilization of psychiatrists and psychologists, which are captured under specialist and allied health categories.

**TABLE 1:** Mean healthcare costs by levels of psychological distress

			Level of psychological distress							
	Full sample		No		Mild		Moderate		Severe	
	Mean	SE	Mean	SE	Mean	SE	Mean	SE	Mean	SE
Panel A: $ of costs										
Total healthcare costs	1197.6	8.9	941.6	11.6	1128.8	18.3	1351.2	23.4	1801.7	25.4
Total MBS costs	952.3	5.4	744.0	6.1	894.4	10.5	1075.9	15.1	1446.7	18.1
Total PBS costs	245.4	6.0	197.6	8.8	234.4	13.2	275.3	15.2	354.9	15.0
GP costs	378.3	1.6	309.6	1.8	362.0	3.1	422.1	4.6	535.8	4.9
Specialist costs	134.6	2.6	82.1	1.9	113.7	4.1	144.2	5.9	283.3	11.3
Allied costs	109.7	1.4	45.3	1.3	96.4	2.9	157.7	4.5	249.9	4.9
Pathology costs	137.4	0.8	124.4	1.0	135.1	1.6	147.7	2.1	165.2	2.0
Number of observations	53,449		24,871		11,008		7745		9825	
Panel B: % with nonzero costs										
Total healthcare costs	97.2		96.7		97.1		97.8		98.2	
Total MBS costs	96.7		96.1		96.7		97.3		97.9	
Total PBS costs	82.3		79.2		81.2		85.1		89.0	
GP costs	94.9		93.9		94.8		95.6		96.8	
Specialist costs	26.7		24.1		26.2		28.2		33.0	
Allied costs	15.9		7.9		14.6		21.9		32.9	
Pathology costs	72.0		69.7		71.8		74.0		76.2	
Panel C: $ of costs conditional on nonzero cost										
Total healthcare costs	1232.0	9.1	973.8	12.0	1162.4	18.7	1382.3	23.8	1834.7	25.8
Total MBS costs	984.6	5.6	774.3	6.2	925.2	10.7	1105.6	15.4	1477.7	18.3
Total PBS costs	298.2	7.3	249.4	11.0	288.6	16.2	323.5	17.8	399.0	16.8
GP costs	398.7	1.6	329.5	1.9	381.9	3.1	441.6	4.6	553.5	4.9
Specialist costs	503.3	8.9	341.1	7.1	434.1	14.0	511.5	18.8	859.2	31.9
Allied costs	690.1	6.0	575.4	11.4	660.7	13.3	719.7	13.4	758.5	10.1
Pathology costs	190.9	0.9	178.3	1.3	188.0	1.9	199.6	2.5	216.7	2.4

*Note*: Total healthcare costs includes both MBS and PBS costs. All costs are in 2019 Australian dollars.

Abbreviations: GP, general practitioners; MBS, Medicare Benefits Schedule; PBS, Pharmaceutical Benefits Scheme.

Panel B reports the proportion of women with non‐zero spending on Medicare services. Based on the full sample, in any given year, 97% of the women aged 18–27 have positive spending. Young women are more likely to use MBS services than PBS, since 97% of the women have positive MBS costs, while only 82% incur PBS costs. A substantial proportion of women do not incur any annual costs on specialist and allied health services, and this is particularly pronounced at the lower levels of psychological distress. When considering the women who have had positive spending (Panel C), the mean total Medicare cost is $1232. For specific MBS items, the mean cost is highest for allied health attendances ($690) followed by specialist attendances ($503).

### 2.4 Covariates

To control for potential confounding factors, we consider several covariates that are commonly used in estimating the healthcare costs of mental disorders. This includes age, highest level of education (indicator variables for grade 10 and below, grade 11, grade 12, certificate, diploma, degree and post‐graduate), employment status (unemployed or not unemployed), income (proxied by the ability to manage income and access to government healthcare card) and marital status (never married, married, de‐facto, separated and divorced) (Bodden et al., 2018; Hsieh & Qin, 2018; Lucas et al., 2013).^7^ We also consider regional and state heterogeneity, by including residence and state dummy variables. Finally, we include year dummies in all the regression models.

Descriptive statistics are presented in Table S2. The average age is 22 years and the majority of the women (70%) are not married. Thirty‐five percent have either a degree or a post‐graduate qualification, which is comparable to national estimates (ABS, 2020a). Approximately 75% of the women live in a metro area, compared to regional and rural, and this is a broad representative of the geographical distribution of the population (Mishra et al., 2014). Almost 30% have access to a healthcare card. Table S2 also displays the descriptive statistics by each level of psychological distress. Women with moderate and severe psychological distress are on average less educated, more likely to be unemployed and find it difficult to manage income, compared to women with no psychological distress, indicating the socioeconomic gradient in mental health (Byles et al., 2012; Hammarström et al., 2011; Kosidou et al., 2011).

## 3 METHODOLOGY

The classical two‐part model of healthcare costs (Jones, 2000; Mullahy, 1998) forms the basis of our empirical model. The first part of the model estimates the probability of incurring positive healthcare costs (extensive margin), while the second part estimates the effect on costs, conditional on incurring any (intensive margin).^8^ The model is specified as;

(1) $$PositiveCosts_{it}=\alpha_{1}+\beta_{1}K10_{it}+\eta_{1}X_{it}^{\prime }+\varphi_{1i}+\gamma_{1t}+\varepsilon_{1\mathrm{it}}$$

(2) $$LogCosts_{it}|PositiveCosts_{it}>0=\alpha_{2}+\beta_{2}K10_{it}+\eta_{2}X_{it}^{\prime }+\varphi_{2i}+\gamma_{2i}+\varepsilon_{2\mathrm{it}}$$

where PositiveCosts_*it*_ takes the value of 1 if woman *i* has non‐zero costs for Medicare services in year *t* and 0 if the woman has zero costs. LogCosts_*it*_ is the logarithm of healthcare costs incurred by woman *i* in year *t*. *K*10_*it*_ is the measure of psychological distress based on K10 score for each women woman *i* in year *t*. We estimate separate models considering K10 score as a continuous variable and dummy variables for each level of psychological distress (i.e., mild, moderate and severe) with no psychological distress as reference category. **X**_*it*_ is a vector of observable characteristics representing age, education, employment status, income, marital status and residence of woman *i* in year *t*. *φ*_*i*_ and *γ*_*t*_ denote unobservable individual and year fixed effects respectively, and *ɛ*_*it*_ is the error term. Throughout the analysis, both equations are specified as linear panel data models, and standard errors are clustered at the individual level and adjusted for heteroskedasticity.

A key strength of the above model is the inclusion of *φ*_*i*_ which captures all unobserved time‐invariant individual‐specific effects that are correlated with psychological distress. Some examples are, systematic heterogeneity in the reporting of mental health (Bago d’Uva et al., 2008), genetic predisposition (Arango et al., 2018) or habits such as substance misuse and addiction that are correlated with distress (American Psychiatric Association, 2013) but are unobserved. The use of fixed‐effect (or within) estimator (FE) eliminates these sources of bias when estimating *β*—the effect of psychological distress on Medicare costs.

However, one caveat of the FE method is that it can lead to biased estimates in the presence of time‐variant unobserved heterogeneity. This may include financial wellbeing, level of health literacy and social stigma, which are associated with both psychological distress and utilization of healthcare services, causing omitted variable bias. To explore the extent of bias caused by selection on such unobservables, we apply the method of Oster (2019). Considering both coefficient and *R*‐squared movements, this approach allows to calculate the degree of proportionality between observed and unobserved variables denoted as delta (*δ*), and thereby estimate the bias‐adjusted effect of psychological distress on healthcare costs. In principle, *δ* = 1 suggests equal importance of both observables and unobservables. If *δ* is closer to zero the unobserved factors are less important than the observed factors, indicating the stability of coefficients (Oster, 2019).

## 4 EMPIRICAL RESULTS

### 4.1 The effect of psychological distress on healthcare costs

We first examine the effect of psychological distress on total healthcare costs (or Medicare costs) which is the summation of annual costs incurred on both medical services (MBS) and pharmaceuticals (PBS). Table 2 presents the empirical results based on the two‐part model (Equations 1 and 2). Columns (1) and (2) report the coefficient estimates from the linear individual fixed‐effects model. The effect of K10 score on the probability of incurring any Medicare costs is positive and significant. With regard to the amount of healthcare cost, an increase in K10 score by one unit increases the conditional cost by 1.6% (column 2). Considering an indicator variable for psychological distress, compared to women with no psychological distress, those with psychological distress have 15% higher healthcare costs (column 2 of Panel B). Panel C shows that the effect of psychological distress on costs increases substantially with each level of symptoms. Specifically, women with moderate psychological distress have 15% higher healthcare costs, than women with no psychological distress. The effect is much larger for women with severe psychological distress, where the conditional cost increases by 26% (column 2 of Panel C). All coefficients are statistically significant at 1%.

**TABLE 2:** Coefficient estimates of the effects of psychological distress on healthcare cost

	Fixed‐effects (FE)		Random‐effects (RE)		OLS	
	(1)	(2)	(3)	(4)	(5)	(6)
	Positive costs	Log costs	Positive costs	Log costs	Positive costs	Log costs
Panel A						
K10 score	0.0003**	0.0157***	0.0006***	0.0244***	0.0007***	0.0315***
	(0.000)	(0.001)	(0.000)	(0.001)	(0.000)	(0.001)
Observations	53,449	51,957	53,449	51,957	53,449	51,957
Panel B						
Psychological distress (=1 if K10 > 24)	0.0050**	0.1517***	0.0097***	0.2824***	0.0108***	0.4345***
	(0.002)	(0.012)	(0.002)	(0.011)	(0.002)	(0.013)
Observations	53,449	51,957	53,449	51,957	53,449	51,957
Panel C						
Ref. group: No psychological distress						
Mild	0.0016	0.0796***	0.0029	0.1310***	0.0036*	0.1756***
	(0.002)	(0.013)	(0.002)	(0.011)	(0.002)	(0.014)
Moderate	0.0066**	0.1522***	0.0093***	0.2494***	0.0096***	0.3382***
	(0.003)	(0.016)	(0.002)	(0.013)	(0.002)	(0.017)
Severe	0.0049	0.2637***	0.0124***	0.4476***	0.0140***	0.6268***
	(0.003)	(0.018)	(0.002)	(0.014)	(0.002)	(0.018)
Observations	53,449	51,957	53,449	51,957	53,449	51,957

*Note*: Robust standard errors in parenthesis, clustered at individual level. All regression models control for age, marital status, education, employment, income management, access to healthcare card, residence, state and year dummies. Positive costs denote the extensive margin of healthcare costs, while log costs denote the intensive margin, conditional on incurring any healthcare costs.

***p < 0.01, **p < 0.05, *p < 0.1.

For comparison, the random‐effects (RE) estimates of the two‐part model are reported in columns (3) and (4) respectively. Similar to within‐individual fixed‐effects model, the effects on both the extensive and intensive margin of healthcare costs are positive and highly significant. However, the effects are much larger in size to the FE estimates. For further comparison, we present the OLS estimates in columns (5) and (6), which are considerably larger than both FE and RE estimates. The smaller FE estimates compared to OLS and RE estimates suggest the presence of unobserved confounders at the individual level, causing an over‐utilization of healthcare services (positive selection bias). This may be due to several reasons such as; (1) positive personality traits, attitudes and habits towards a healthy lifestyle which are correlated with a lower likelihood of psychological distress and a higher use of healthcare services (due to regular medical check‐ups and health screening), (2) family history of mental health problems (genetic endowments) leading to both higher likelihood of distress and healthcare costs, (3) high health expectations causing young women to assess a health condition negatively (Bago d’Uva et al., 2008), and thereby a greater use of healthcare services.^9^

The actual marginal/incremental healthcare costs of psychological distress are shown in Table 3. The conditional predicted costs in dollar scale are estimated from equation two of the two‐part model. The unconditional predicted costs are derived by multiplying the predicted probabilities and costs from each part of the two‐part model. We apply the Duan (1983) smearing estimator to transform predictions of log expenditures into its levels. Since healthcare costs vary by the levels of distress, we use separate smearing factor by K10 scores to account for its heteroskedasticity (Manning & Mullahy, 2001). On average, a one‐unit increase in K10 score increases annual unconditional healthcare costs by $18. Considering an indicator variable for psychological distress, on average, women with psychological distress have higher total healthcare costs of $179 than women with no psychological distress (Panel B column 2). As seen in Table 2, the incremental unconditional effect of psychological distress on costs increases with the severity of distress. In particular, women with mild, moderate or severe distress have higher healthcare costs of $93, $179 and $310 than women with no symptoms of distress respectively. Tables 1 and 2 indicate that the higher healthcare costs of psychological distress are primarily due to an increase in spending by women with positive spending, rather than an increase in the probability of spending. This is plausible given that almost 97% of women have a positive cost on healthcare services.

**TABLE 3:** Conditional and unconditional marginal/incremental effects of psychological distress on healthcare cost (dollars)

	Fixed‐effects (FE)		Random‐effects (RE)		OLS	
	(1)	(2)	(3)	(4)	(5)	(6)
	Conditional costs ($)	Unconditional costs ($)	Conditional costs ($)	Unconditional costs ($)	Conditional costs ($)	Unconditional costs ($)
Panel A						
K10 score (BS)	19.53***	18.28***	29.83***	21.95**	38.77***	23.53*
	(1.22)	(4.99)	(0.80)	(10.13)	(0.84)	(13.82)
Panel B						
Psychological distress	188.24***	178.71***	345.73***	248.55**	535.39***	327.26*
(1 if K10 > 24)	(15.15)	(60.15)	(11.05)	(118.23)	(13.05)	(194.04)
Panel C						
Ref. group: No psychological distress						
Mild	98.78***	93.14***	160.34	116.28**	216.07***	131.10*
	(17.85)	(31.51)	(14.21)	(54.45)	(12.88)	(77.19)
Moderate	188.93***	179.03***	305.23	222.76**	416.24***	254.07*
	(20.03)	(60.56)	(14.76)	(104.31)	(16.67)	(149.60)
Severe	327.28***	309.60***	547.92	401.11**	771.39***	472.90*
	(22.65)	(104.73)	(17.09)	(187.83)	(18.99)	(278.46)

*Note*: The conditional costs are the marginal/incremental effects in dollar scale estimated from the second part of the model. The unconditional predicted costs are derived by multiplying the predicted probabilities and costs from each part of the two‐part model. Bootstrapped standard errors in parenthesis. Duan smearing factor by K10 scores is used to transform predictions of log expenditures into its levels. All costs are in 2019 Australian dollars (AUD) and adjusted for inflation.

***p < 0.01, **p < 0.05, *p < 0.1.

### 4.2 Addressing potential omitted variable bias

The FE estimates reported in Table 2 may be biased if there is selection on unobservables. To assess the extent of bias caused by unobservable characteristics we use Oster (2019) method. Panel B of Table 4 reports the results. The baseline effect (FE estimate without controls) and controlled effect (FE estimate with all controls) are quite similar, implying that once we account for time‐invariant heterogeneity there is minimum time‐varying selection bias. This is further reassured by the magnitude of delta (*δ*). For both measures of psychological distress (K10 score and distress dummy), values of *δ* are almost zero under both *R*_max_ assumptions.^10^ Further, the bias‐adjusted effects assuming *δ* = 1 are identical to the FE estimates. This suggests that the unobservables or omitted variables are less influential in explaining the effect of psychological distress on healthcare costs than the included controls.

**TABLE 4:** Estimates from Oster method

	(1)	(2)	(3)		(4)	
	Baseline effect $\left(\dot{\beta }\right)$	Controlled effect $\left(\tilde{\beta }\right)$	Delta (*δ*) for estimated $\tilde{\beta }$ given:		Bias adjusted effect for *δ* = 1 given:	
	(SE) [*R* ^2^]	(SE) [*R* ^2^]	*R*_max_ = 1.3$\tilde{R}$	*R*_max_ = 2.2$\tilde{R}$	*R*_max_ = 1.3$\tilde{R}$	*R*_max_ = 2.2$\tilde{R}$
K10 score	0.013	0.016	0.245	0.059	0.017	0.020
	(0.001) [0.006]	(0.001) [0.025]				
Psychological distress	0.132	0.152	0.021	0.007	0.166	0.193
	(0.012) [ 0.003]	(0.012) [ 0.021]				

*Note*: Robust standard errors in parenthesis, clustered at individual level. Controls include age, marital status, education, employment, income management, access to healthcare card, residence, state and year dummies. The estimates on conditional costs obtained from the linear model of log costs are reported. *N* = 51,957.

***p < 0.01, **p < 0.05, *p < 0.1.

Overall, the results in Table 4 imply that omitted variable bias is not likely to bias the FE estimates. Therefore, we proceed with the two‐part model with individual fixed‐effects as our preferred model of estimation for the remaining analysis.

### 4.3 Heterogeneity by type of healthcare costs

In this section, we investigate whether the effect of psychological distress is heterogeneous by main types of Medicare cost; health services use (MBS) and pharmaceuticals (PBS). Table 5 reports the FE coefficient estimates from the two‐part model. An increase in K10 score by one unit increases the conditional MBS and PBS costs by 1.6% and 0.9% respectively. Considering indicator variables, young women with moderate and severe psychological distress have 16 and 28% higher costs on MBS medical services respectively, than women with no psychological distress. The estimates are smaller for PBS services, as moderate and severe psychological distress lead to a corresponding increase of 8 and 14%. Table 6 presents the marginal/incremental effects of psychological distress on MBS and PBS costs. The unconditional costs on MBS and PBS increase by $13 and $2 respectively as the K10 score increases by one unit. Moreover, women with moderate and severe psychological distress incur $128 and $226 higher unconditional costs on MBS medical services, which is considerably larger than costs on PBS medications. These findings imply that the use of MBS medical services contributes to a higher proportion of total healthcare costs of psychological distress.

**TABLE 5:** Coefficient estimates of the effects of psychological distress by type of Medicare cost

	MBS		PBS	
	(1)	(2)	(3)	(4)
	Positive costs	Log costs	Positive costs	Log costs
Panel A				
K10 score	0.0004**	0.0164***	0.0014***	0.0094***
	(0.000)	(0.001)	(0.000)	(0.001)
Observations	53,449	51,696	53,449	43,978
Panel B				
Ref. group: No psychological distress				
Mild	0.0034	0.0840***	0.0008	0.0346**
	(0.003)	(0.013)	(0.005)	(0.017)
Moderate	0.0077***	0.1576***	0.0139**	0.0754***
	(0.003)	(0.016)	(0.006)	(0.020)
Severe	0.0057*	0.2791***	0.0213***	0.1415***
	(0.003)	(0.018)	(0.007)	(0.023)
Observations	53,449	51,696	53,449	43,978

*Note*: Robust standard errors in parenthesis, clustered at individual level. All regression models control for age, marital status, education, employment, income management, access to healthcare card, residence, state and year dummies and individual fixed‐effects. Positive costs denote the extensive margin of healthcare costs, while log costs denote the intensive margin, conditional on incurring any healthcare costs.

Abbreviations: MBS, Medicare Benefits Schedule; PBS, Pharmaceutical Benefits Scheme.

***p < 0.01, **p < 0.05, *p < 0.1.

**TABLE 6:** Conditional and unconditional marginal/incremental effects of psychological distress by type of Medicare cost (dollars)

	MBS		PBS	
	(1)	(2)	(3)	(4)
	Conditional costs ($)	Unconditional costs ($)	Conditional costs ($)	Unconditional costs ($)
Panel A				
K10 score	16.22***	13.09**	2.84***	1.65*
	(1.04)	(5.05)	(0.37)	(0.88)
Panel B				
Ref. group: No psychological distress				
Mild	83.19***	67.77**	10.41*	6.07*
	(16.39)	(25.93)	(5.52)	(3.21)
Moderate	156.05***	127.66**	22.69***	13.40*
	(18.14)	(48.84)	(7.23)	(7.09)
Severe	276.35***	225.63**	42.61***	25.34*
	(20.46)	(86.32)	(8.20)	(13.41)

*Note*: The conditional costs are the marginal/incremental effects in dollar scale estimated from the second part of the model. The unconditional predicted costs are derived by multiplying the predicted probabilities and costs from each part of the two‐part model. Bootstrapped standard errors in parenthesis. Duan smearing factor by K10 scores is used to transform predictions of log expenditures into its levels. All costs are in 2019 Australian dollars (AUD) and adjusted for inflation.

Abbreviations: MBS, Medicare Benefits Schedule; PBS, Pharmaceutical Benefits Scheme.

***p < 0.01, **p < 0.05, *p < 0.1.

Given the higher use of MBS medical services, it is useful to examine which specific MBS categories drive the total healthcare costs of psychological distress. The FE coefficient estimates presented in Table 7 indicate that women with psychological distress have higher costs on allied attendances, followed by specialist and GP attendances. Specifically, a one unit increase in K10 score increases the conditional costs of allied, specialist and GP visits by 2.3%, 1.4% and 1.2% respectively. A similar pattern is observed even if we use indicator variables to denote levels of psychological distress (Panel B). Women with severe psychological distress have 41% higher conditional costs on allied attendances than women with no psychological distress, which is nearly double the additional costs they incur on specialist (25%) and GP attendances (21%). However, when considering the marginal/incremental effects in Table 8 we observe that compared to conditional costs, the unconditional costs specifically on allied health and specialist attendances are smaller. This is because a substantial proportion of women (more than 75%) have zero positive costs on these services. Moreover, as we would expect, both K10 score and the dummy variables for psychological distress do not have a significant effect on the conditional costs of other MBS categories such as imaging, operations, optometry and other services, as reported in Table S3.

**TABLE 7:** Coefficient estimates of the effects of psychological distress on various MBS items

	GP		Specialist		Allied		Pathology	
	(1)	(2)	(3)	(4)	(5)	(6)	(7)	(8)
	Positive costs	Log costs	Positive costs	Log costs	Positive costs	Log costs	Positive costs	Log costs
Panel A								
K10 score	0.0006***	0.0124***	0.0022***	0.0140***	0.0093***	0.0232***	0.0007	0.0036***
	(0.000)	(0.001)	(0.000)	(0.002)	(0.000)	(0.002)	(0.000)	(0.001)
Observations	53,449	50,706	53,449	14,286	53,449	8490	53,449	38,468
Panel B								
Ref. group: No psychological distress								
Mild	0.0045	0.0584***	0.0131**	0.0935***	0.0421***	0.1475***	0.0030	0.0147
	(0.003)	(0.011)	(0.006)	(0.030)	(0.005)	(0.044)	(0.006)	(0.015)
Moderate	0.0068*	0.1172***	0.0136*	0.1399***	0.0852***	0.3091***	0.0134*	0.0305*
	(0.004)	(0.013)	(0.007)	(0.038)	(0.006)	(0.044)	(0.008)	(0.018)
Severe	0.0102**	0.2093***	0.0351***	0.2539***	0.1653***	0.4077***	0.0068	0.0504**
	(0.004)	(0.015)	(0.008)	(0.041)	(0.008)	(0.045)	(0.009)	(0.020)
Observations	53,449	50,706	53,449	14,286	53,449	8490	53,449	38,468

*Note*: Robust standard errors in parenthesis, clustered at individual level. All regression models control for age, marital status, education, employment, income management, access to healthcare card, residence, state and year dummies and individual fixed‐effects. Positive costs denote the extensive margin of healthcare costs, while log costs denote the intensive margin, conditional on incurring any healthcare costs.

Abbreviations: GP, general practitioners; MBS, Medicare Benefits Schedule.

***p < 0.01, **p < 0.05, *p < 0.1.

**TABLE 8:** Conditional and unconditional marginal/incremental effects of psychological distress on various MBS items (dollars)

	GP		Specialist		Allied	
	(1)	(2)	(3)	(4)	(5)	(6)
	Conditional costs ($)	Unconditional costs ($)	Conditional costs ($)	Unconditional costs ($)	Conditional costs ($)	Unconditional costs ($)
Panel A						
K10 score	4.94***	4.54***	7.13***	5.03	16.22***	7.30**
	(0.29)	(1.20)	(1.62)	(4.50)	(1.90)	(3.24)
Panel B						
Ref. group: No psychological distress						
Mild	23.31***	21.49***	47.57**	32.75**	103.03**	49.22**
	(4.36)	(5.69)	(22.11)	(15.28)	(37.35)	(20.53)
Moderate	46.79***	43.23***	71.17***	47.99**	215.95***	107.43**
	(5.05)	(11.45)	(24.01)	(22.39)	(34.11)	(44.81)
Severe	83.55***	77.46***	129.21***	88.60**	284.83***	152.15**
	(5.75)	(20.51)	(30.59)	(41.33)	(41.88)	(63.47)

*Note*: The conditional costs are the marginal/incremental effects in dollar scale estimated from the second part of the model. The unconditional predicted costs are derived by multiplying the predicted probabilities and costs from each part of the two‐part model. Bootstrapped standard errors in parenthesis. Duan smearing factor by K10 scores is used to transform predictions of log expenditures into its levels. All costs are in 2019 Australian dollars (AUD) and adjusted for inflation.

Abbreviations: GP, general practitioners; MBS, Medicare Benefits Schedule.

***p < 0.01, **p < 0.05, *p < 0.1.

### 4.4 Heterogeneity by onset and chronicity of psychological distress

We now examine whether the onset of psychological distress will have a different effect to having psychological distress on healthcare costs. We define onset of psychological distress as the first reporting of psychological distress (i.e., a K10 score >24) preceded by no psychological distress in previous waves (Pan et al., 2020). For this purpose, we first restrict our sample to a balanced panel. Next, we drop those women who have psychological distress in all five waves and women with psychological distress in the first wave. To capture chronicity of psychological distress, we create dummy variables based on the number of times the women have psychological distress throughout the five waves. That is we estimate three alternate models using psychological distress defined as only if they have it in (1) at least 2 waves, (2) at least 3 waves, and (3) in all four waves. Fixed‐effects estimates reported in Table 9 show that an onset of psychological distress (at least in one wave) increases the healthcare costs by 17%. This is slightly larger than the baseline estimate of 15% from the complete unbalanced sample reported in Table 2. As anticipated, persistent psychological distress leads to higher conditional healthcare costs. Specifically, women with psychological distress in at least 2, 3 and all 4 waves have 21%, 28% and 25% higher healthcare costs respectively, than women with no psychological distress.^11^

**TABLE 9:** Coefficient estimates of the effects of onset and chronicity of psychological distress on healthcare costs

	At least in 1 wave		At least in 2 waves		At least in 3 waves		All 4 waves	
	(1)	(2)	(3)	(4)	(5)	(6)	(7)	(8)
	Positive costs	Log costs	Positive costs	Log costs	Positive costs	Log costs	Positive costs	Log costs
Psychological distress	0.0107**	0.1745***	0.0057	0.2121***	0.0125	0.2814***	0.0219	0.2457**
	(0.004)	(0.025)	(0.006)	(0.033)	(0.011)	(0.051)	(0.020)	(0.113)
Observations	17,945	17,371	17,945	17,371	17,945	17,371	17,945	17,371
% with distress	12%		9%		5%		2%	

*Note*: Robust standard errors in parenthesis, clustered at individual level. All regression models control for age, marital status, education, employment, income management, access to healthcare card, residence, state and year dummies and individual fixed‐effects. Positive costs denote the extensive margin of healthcare costs, while log costs denote the intensive margin, conditional on incurring any healthcare costs.

***p < 0.01, **p < 0.05, *p < 0.1.

### 4.5 Monthly variations and dynamics in healthcare costs

The measurement of psychological distress based on the K10 score can reflect a short‐term phenomenon as it reports on the symptoms during the past four weeks (Lebenbaum et al., 2021). This reference period is much shorter than the reference period for the outcome of interest (annual healthcare costs), resulting in a timing mismatch. As this is likely to underestimate the true effect of psychological distress on healthcare costs, we investigate whether the effect varies with the time‐period under consideration. In this regard, we estimate the effect on derived monthly healthcare costs by using the month of the survey returned of each individual as the starting point. For instance, if a woman has returned the survey during the month of May in a given year, we match the Medicare records of that particular month to estimate the total costs incurred during the first month (T1) since the occurrence of psychological distress. The total costs for 2 months (T2) is obtained by adding the costs incurred in May and June while the total cost for 3 months (T3) is the summation of costs in May, June and July. We follow this pattern until T12, which represents the total costs for the period of 12 months from the survey returned month.

**FIGURE 3:**
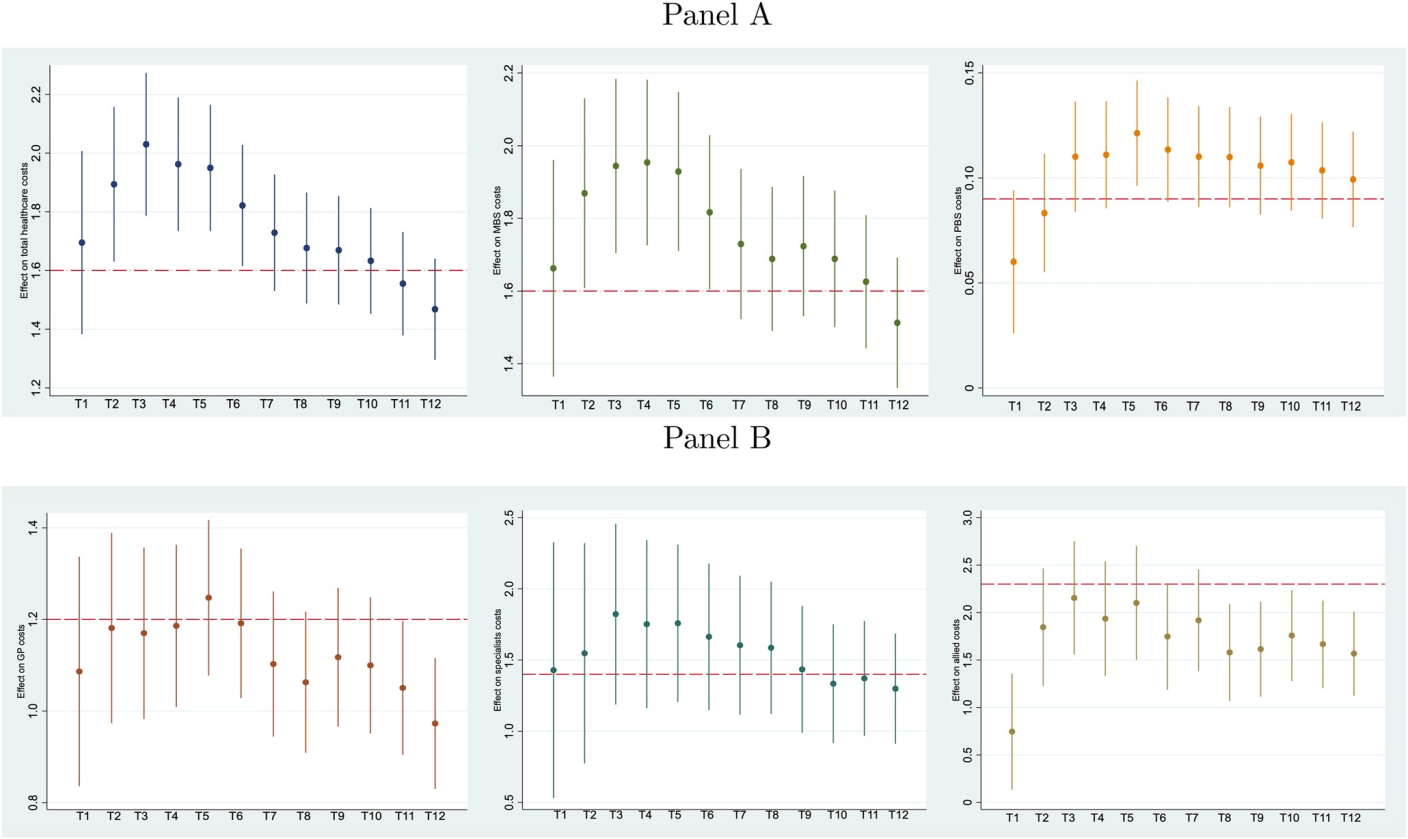
Monthly variation in healthcare costs. The effects represent the estimates obtained from the second part of the model on conditional monthly costs, which are statistically significant at 1% level. All estimations control for age, marital status, education, employment, income management, access to healthcare card, residence, state and time dummies and individual fixed‐effects. The dashed lines indicate the corresponding estimates obtained from the two‐part model on conditional annual costs.

The estimates from the two‐part model on derived monthly healthcare costs are shown in Table S4 and the effects of K10 score on conditional costs are summarized graphically in Figure 3. According to Panel A, the effect of K10 score on total healthcare costs is highest at the 3 month period (about 2%) which then decreases gradually to 1.5% at the 12‐months period. On average these effects tend to vary within the range of 1.5–2.0% and are quite similar to the estimate from the FE model on annual costs (1.6%) indicated by the dashed line. We observe a similar pattern for the two types of Medicare costs; MBS and PBS services, where the effect of psychological distress tend to be highest during the first 6 months compared to the last 6 months since reporting of symptoms.

Panel B illustrates the effects of K10 score on monthly healthcare costs by types of specific MBS categories. When considering costs on allied attendances, there is a substantial spike in the effect from T1 to T2 period, which then fluctuates within the range of 1.8–2.3%. The effects on costs of specialist attendances also show an oscillating pattern, which is highest during the period of three to 5 months since onset. Given that the monthly effects on average are very much similar to the estimates obtained from the annual costs, we believe there is minimum measurement error bias due to timing mismatch of reporting of psychological distress and healthcare costs.

### 4.6 The effect of psychological distress on out‐of‐pocket healthcare costs

We now turn to estimate the effect of psychological distress on out‐of‐pocket (OOP) healthcare costs, as previous studies show a higher burden among those with depression (Callander et al., 2017; Harman et al., 2004; Rajan et al., 2020). Table 10 presents the FE estimates from the two‐part model. A one‐unit increase in K10 score increases the conditional OOP costs on total healthcare services by 1.5%. We further observe that women with psychological distress have higher OOP expenditure on medical services covered by MBS (1.7%) than on PBS pharmaceuticals (1%). In terms of the indicator variable, young women experiencing psychological distress have 16 and 8% higher OOP costs on MBS and PBS services respectively, than women who do not experience psychological distress.

**TABLE 10:** Coefficient estimates of the effects of psychological distress on out‐of‐pocket healthcare costs

	Out‐of‐pocket costs on					
	Total healthcare services		MBS services		PBS medicines	
	(1)	(2)	(3)	(4)	(5)	(6)
	Positive costs	Log costs	Positive costs	Log costs	Positive costs	Log costs
Panel A						
K10	0.0010***	0.0149***	0.0024***	0.0177***	0.0014***	0.0100***
	(0.000)	(0.001)	(0.000)	(0.001)	(0.000)	(0.001)
Observations	53,449	47,739	53,449	31,138	53,449	43,929
Panel B						
Psychological distress	0.0146***	0.1222***	0.0208***	0.1616***	0.0164***	0.0807***
	(0.004)	(0.015)	(0.006)	(0.020)	(0.005)	(0.013)
Observations	53,449	47,739	53,449	31,138	53,449	43,929

*Note*: Robust standard errors in parenthesis, clustered at individual level. All regression models control for age, marital status, education, employment, income management, access to healthcare card, residence, state and year dummies and individual fixed‐effects. Positive costs denote the extensive margin of out‐of‐pocket healthcare costs, while log costs denote the intensive margin, conditional on incurring any out‐of‐pocket healthcare costs.

***p < 0.01, **p < 0.05, *p < 0.1.

Considering higher OOP costs on MBS medical services, we investigate whether these OOP costs are driven by mental health specific services or other medical services. Table 11 shows that women with psychological distress have higher OOP costs for mental health‐related allied services (such as clinical psychologists, psychologists, and occupational therapists) and specialist services (consultant psychiatrists). A one unit increase in K10 score increases the total OOP costs on allied and specialist mental health related services by 2.1% and 1.6% respectively. Interestingly, there is no statistically significant effect on OOP costs related to other (non mental health specific) allied and specialist services. The findings are consistent even if we use an indicator variable to denote psychological distress. Women experiencing psychological distress have 21% higher OOP costs for allied and specialist mental health related services, than women with no psychological distress. Overall, this indicates that mental health related services are the main source of higher OOP costs among women with psychological distress.^12^

**TABLE 11:** Coefficient estimates of the effects of psychological distress on mental health and non‐mental health specific out‐of‐pocket healthcare costs

	GP				Specialist items			
	Mental health (GP mental health items)		Non‐mental health (Other GP items)		Mental health (Consultant Psychiatrist)		Non‐mental health (Other specialist items)	
	(1)	(2)	(3)	(4)	(5)	(6)	(7)	(8)
	Positive costs	Log costs	Positive costs	Log costs	Positive costs	Log costs	Positive costs	Log costs
Panel A								
K10	0.0023***	0.0102	0.0002	0.0071***	0.0012***	0.0139**	0.0016***	0.0021
	(0.000)	(0.007)	(0.000)	(0.001)	(0.000)	(0.006)	(0.000)	(0.003)
Observations	53,449	1355	53,449	24,056	53,449	1386	53,449	9701
Panel B								
Psychological distress	0.0246***	−0.0538	0.0008	0.0427**	0.0123***	0.2115**	0.0095*	−0.0159
	(0.003)	(0.105)	(0.006)	(0.018)	(0.002)	(0.089)	(0.005)	(0.038)
Observations	53,449	1355	53,449	24,056	53,449	1386	53,449	9701

	Allied health			
	Mental health (Clinical psychologist, psychologist, occupational therapist and social worker)		Non‐mental health (Other allied health items)	
	(9)	(10)	(11)	(12)
	Positive costs	Log costs	Positive costs	Log costs
Panel A				
K10	0.0058***	0.0211***	0.0002	−0.0142
	(0.000)	(0.003)	(0.000)	(0.016)
Observations	53,449	4218	53,449	623
Panel B				
Psychological distress	0.0606***	0.2146***	−0.0008	−0.1421
	(0.004)	(0.050)	(0.002)	(0.140)
Observations	53,449	4218	53,449	623

### 4.7 Aggregate costs attributable to psychological distress among young women

Based on our predicted unconditional incremental effects from the FE model, we extrapolate the results to the Australian population to calculate the aggregate costs attributable to psychological distress among young women. There are approximately 1.72 million women aged 18–27 years in Australia (ABS, 2020b), of whom about 21% have mild psychological distress, 14% have moderate psychological distress and 18% have severe psychological distress (estimates from ALSWH). Table 12 reports the extrapolated cost estimates, which we obtain by multiplying the average predicted extra cost per woman in each level of psychological distress (as shown in Table 3, Panel C Column 2) by the total population of mild, moderate and severe psychological distress. We find that for women aged 18–27 years, compared to women with no psychological distress, the additional healthcare cost attributable to mild, moderate and severe psychological distress is approximately $33.66 million, $43.19 million and $96.17 million respectively.

**TABLE 12:** Annual healthcare cost attributable to psychological distress among women aged 18–27

	Healthcare cost (in millions of 2019 AUD)	Population
Mild	$33.66	361,925
Moderate	$43.19	241,283
Severe	$96.17	310,222

*Note*: The healthcare costs include both MBS and PBS costs. Average excess cost per woman in each level is from the unconditional incremental effects shown in Table 3.

## 5 ROBUSTNESS CHECKS

We conduct several sensitivity checks to examine the robustness of our findings. First, we use a balanced panel to identify whether our estimates are affected by sample attrition bias. Table S6 reports the results. The coefficients from the balanced panel for both extensive and intensive margin of healthcare costs are slightly larger compared to estimates from the unbalanced panel reported in Table 2. The differences in the magnitude of the effects on conditional costs from the balanced and unbalanced panels are small (approximately 0.1% for the K10 score and two percent for the dummy variable on psychological distress), indicating the estimates are less likely to be affected by potential biases from sample attrition.^13^

Second, we assess the validity of K10 score as a reliable measure of psychological distress. In this regard we consider two alternative measures of depression based on two different questions in ALSWH survey; (1) Have you ever been diagnosed with or treated for depression; and (2) In the last 12 months, have you had depression. Specifically, we estimate two separate equations with a dummy variable for diagnosed/treated for depression (=1 if marked yes, and 0 otherwise) and another dummy variable for depression in last year (=1 if answered sometimes or often, and 0 otherwise). It is important to note that these two questions are not included in Survey 4, and therefore the FE estimates from the two‐part model reported in Table S7 are based on an unbalanced panel created using data from waves 1, 2, 3 and 5. For comparison, we also report the results obtained using a dummy variable for psychological distress based on the K10 score (Panel A, columns 1 and 2). The estimates using dummy variables derived from K10 score and felt depression in the past year are quite similar, as both suggest that women with psychological distress/depression have 15–16% higher costs. However, estimates using the dummy variable based on diagnosed/treated depression show almost a 19% increase in costs. This is expected as diagnosed/treated depression may be of chronic nature when compared to depression based on a symptom scale such as K10 score or depression feelings (Lebenbaum et al., 2021), and thus leading to a higher effect on healthcare costs.

Third, our main model does not include health status or other health conditions because these could be potentially correlated with psychological distress. However, there might be concerns about the validity of the estimates if these conditions have an independent and significant effect on healthcare utilization, causing potential omitted variable biases (Black et al., 2018). To allay this concern, we re‐estimate our two‐part model including indicators for health status and conditions such as low iron, asthma, endometriosis and polycystic ovary syndrome (PCOS).^14^ The estimates reported in Panel B of Table S7 show that even after controlling for these conditions the effect of psychological distress on intensive margin of healthcare costs is still statistically significant at 1% and is quite similar to those reported in Panel A. This provides support that physical health conditions are less likely to drive the effect of psychological distress on healthcare costs.^15^

## 6 DISCUSSION

Mental illness such as depression in young adults is a global health issue leading to health, economic and social consequences (WHO, 2008). While previous studies show a positive association between youth depression and healthcare costs, the magnitude of its impact on healthcare cost remains relatively unexplored (Bodden et al., 2018). This study addresses this important issue, by using nationally representative survey data linked to administrative Medicare records from Australia. The use of national Medicare claims data allows to estimate precise government‐funded healthcare costs attributable to psychological distress.

It is important to note that though our analysis does not fully address the causality claims, we still attempt to address certain key endogeneity issues that are usually prevalent in similar studies. Our preferred identification strategy is based on the classical two‐part model of healthcare costs with individual specific fixed‐effects, where we show that once we account for time‐invariant heterogeneity, there is minimum time‐varying selection bias. We further address the concern of omitted variable bias by applying Oster (2019) method for selection on unobservables. The analysis using monthly data provides suggestive evidence that reverse causality or simultaneity bias is also unlikely to bias our estimates. This is because the measure of healthcare costs is derived based on the months following the reporting of psychological distress. In addition, we also examine the sensitivity of our estimates to attrition bias and measurement error. However, given that we do not account for time‐varying unobservables and simultaneity bias fully, we caution against a strict causal interpretation.

We find that women with moderate psychological distress have 15% higher healthcare costs, than women with no psychological distress. The effect is much larger for women experiencing severe psychological distress, where costs increase by 26%. We further find significant heterogeneity on several dimensions, such as persistence of the psychological distress and short and long‐term effects since the occurrence of the symptoms. To validate our findings, we exploit the richness of the administrative data on Medicare records to investigate whether the higher healthcare costs are in fact driven by the use of mental health related services. To this end, we examine the percentage utilization of specific mental health related MBS and PBS items compared to all other items by levels of psychological distress, which is summarized in Table S8. The use of mental health items increases with each level of psychological distress. Notably, when considering specialist attendance, the highest uptake of consultant psychiatrist items is among the women with severe psychological distress, which is almost 50% of their total specialist attendances. Similarly, out of total allied attendances, almost 84% are either for clinical psychologist or psychologist items coming under the Better Access to Mental Health Care program. We further look at the use of mental health related medications based on the Anatomical Therapeutic Chemical (ATC) codes.^16^ The use of mental health related ATC codes is highest among women with severe psychological distress (44%) followed by women with moderate psychological distress (34%). A larger proportion of the total use is through the use of antidepressants. This provides support that mental health related services are the main source of excess costs of psychological distress in general.

Extrapolating our results to the total population of women aged 18–27 years in Australia indicates that the additional healthcare cost attributable to moderate and severe psychological distress is approximately $139 million. To put this value into perspective, this represents 7% of the Australian government spending of 1966 million on Medicare subsidized mental health specific services and mental health‐related prescriptions under the PBS/RPBS in 2019/20 (AIHW, 2021).

Our study has some data limitations. First, our sample of women (1989–95 cohort of ALSWH) may represent a convenience sample, as women were recruited using the Internet and social media platforms. This suggests that there is potential for selection along observable characterizes such as education and health status. However, Mishra et al. (2014) show that this cohort of women is a broadly representative sample of the Australian population based on a comparison of sociodemographic and health characteristics with comparable Australian Census and Health Survey data. Second, the K10 measure of psychological distress is based on self‐reported assessments, which may lead to measurement error (Frijters et al., 2014; Lebenbaum et al., 2021).^17^ According to Bago d’Uva et al. (2008), young women are more likely to have high health expectations and thereby to assess a health condition negatively, implying an over‐reporting of symptoms. The use of individual fixed‐effects can account for such systematic heterogeneity in the self‐reporting of mental health. To further mitigate the concern of reporting bias, we consider alternative measures of depression based on diagnosed/treated for depression and show that the estimates are quite similar. Third, the Medicare data used in our analysis exclude all hospital costs due to the unavailability of public hospital records. This suggests that the effect of psychological distress on total Government healthcare costs may be underestimated. However, when considering mean differences of costs between women with psychological distress and no psychological distress, the magnitude of the difference is quite similar to other studies that report mean differences of various mental health disorders on total costs including hospital services (Bock et al., 2016; Ride et al., 2019). This implies that the omission of hospital costs is less likely to affect the estimated effect.^18^

Our findings have significant policy implications. The prevalence of psychological distress is increasing, particularly among young adults (Burns et al., 2020; Butterworth et al., 2020; Twenge et al., 2019). Our results show that the excess healthcare costs of psychological distress in young women in Australia are substantial, amounting to AUD 139 million annually. Given that psychological distress can lead to major mental health illnesses such as major depression (Horwath et al., 1992), which further aggravate the burden on healthcare resources (Ride et al., 2019), our findings highlight the importance of policy initiatives aimed at early intervention and prevention. We further find that women with psychological distress incur higher out‐of‐pocket costs on mental healthcare (specifically for services provided by consultant psychiatrists, clinical psychologists and psychologists) than on non‐mental health services. This suggests that higher costs may hinder young individuals from accessing required care, necessitating policy interventions to make mental healthcare affordable, particularly for young women. Such policy measures would inevitably reduce not only the economic consequences of psychological distress, but also the larger costs of other mental illnesses.

## 7 CONCLUSION

The ongoing increase in the prevalence of psychological distress in young adults is a global concern. However, there is limited evidence on the magnitude of this issue in monetary terms. We contribute substantially to this space, by estimating the healthcare costs attributable to psychological distress using Australian longitudinal survey and administrative data. We find that moderate and severe psychological distress are associated with 15 and 26% higher healthcare costs compared to no psychological distress. Policy‐wise, our findings underscore the importance of providing affordable and accessible mental health services, especially targeting young women.

## CONFLICT OF INTEREST

The authors declare that they have no conflicts of interest.

## Supporting information

Supplementary Material

## Data Availability

Data subject to third party restrictions—ALSWH survey data are owned by the Australian Government Department of Health and due to the personal nature of the data collected, release by ALSWH is subject to strict contractual and ethical restrictions. Ethical review of ALSWH is by the Human Research Ethics Committees at The University of Queensland and The University of Newcastle. De‐identified data are available to collaborating researchers where a formal request to make use of the material has been approved by the ALSWH Data Access Committee. The committee is receptive of requests for datasets required to replicate results. Information on applying for ALSWH data is available from https://alswh.org.au/for‐data‐users/applying‐for‐data/. In addition, linked administrative data have been provided by the following third parties; Australian Government Department of Health and Aged Care, and Australian Institute of Health and Welfare (AIHW). In order for these linked data to be accessed through ALSWH, every data user must be added to the applicable Data Use Agreements and Human Research Ethics Committee protocols.

## ENDNOTES

^1^
Direct costs are the costs directly attributable to diagnosis, treatment and care of a health condition. This includes both individual healthcare costs and non‐healthcare costs, such as cost of support services or disability payments (Bloom et al., 2011).

^2^
There were 17,010 women in the baseline 2013 survey, and out of these 22% are observed only in wave 1. We find that attrition is positively associated with K10 score, that is those with psychological distress are more likely to drop out of the sample. This suggests attrition bias is likely to underestimate the effect of psychological distress on healthcare costs. We examine this using a balanced panel as a robustness check.

^3^
Please see Table S1 in Supplementary materials for the sample questionnaire.

^4^
According to Kessler et al. (1992), there is a quadratic relationship between age and depressive symptoms. Specifically, the depressive symptoms are highest in the youngest and oldest adults, yielding a *U*‐shaped curve. This is also confirmed by studies such as Akhtar‐Danesh and Landeen (2007); Mirowsky and Reynolds (2000) and Schieman et al. (2002). This suggests that the decline in the prevalence of depressive symptoms with age is likely to reflect age‐based differences rather than a reduction in the incidence of mental illness.

^5^
This initiative aims to enhance accessibility and affordability of professional mental health care services to improve outcomes for patients with mental health disorders. See Department of Health and Ageing (2010) for further details.

^6^
This includes both the Medicare benefit paid by the government and the out‐of‐pocket cost.

^7^
The survey data from the 1989–95 cohort of ALSWH does not contain any income variable. Therefore we use categorical dummies for management of income (impossible, difficult and easy) and a government healthcare card dummy as proxies for income status. Since this may cause potential omitted variable bias, we follow Oster (2019) to estimate the unbiased effect of psychological distress on healthcare costs in the presence of such unobserved confounders, discussed under Section 3.

^8^
It is important to note that the log‐linear model was a better‐fit when compared to generalized linear models (GLMs). We compared the log‐linear model to several GLM models (such as GLMs with gamma distributions with log, power(0.5) and power(0.1) links) by using a variety of goodness‐of‐fit measures. This includes skewness and kurtosis of the residuals, Pearson's correlation test, Pregibon's Link test, Ramsey RESET test, mean prediction error, mean absolute prediction error and root mean square error.

^9^
We conducted a Hausman test which strongly rejected the null hypothesis that the unobserved heterogeneity is not correlated with the other regressors, suggesting FE estimator is preferred over RE.

^10^
It is possible to consider various values for *R*_max_. In our analysis we follow Oster (2019) and Hener et al. (2016) in setting the *R*_max_ = 1.3$\tilde{R}$ and *R*_max_ = 2.2$\tilde{R}$ respectively.

^11^
The coefficient estimates reported in columns 7 and 8 may should be interpreted with caution since the percentage of women with psychological distress in all four waves is only 2% leading to low statistical power.

^12^
Table S5 reports the marginal/incremental effects for the out‐of‐pocket (OOP) estimates. Despite higher conditional OOP costs for specialist and allied mental health related services, the unconditional OOP costs are considerably lower. This is because only a small proportion of women have positive OOP spending on these services, which is 2.6% and 7.9% respectively.

^13^
From an economic sense, attrition does not cause serious bias, even when there is large sample attrition (Cheng & Trivedi, 2015).

^14^
These health conditions are also not reported in Survey 4, and therefore we use panel data from waves 1, 2, 3 and 5. The health status is denoted as a dummy variable which is assigned one if reported either excellent, very good or good for the survey question, “In general, would you say your health is”, and zero otherwise (i.e., fair and poor).

^15^
We only control for a selected number of physical health conditions since data on other major conditions such as heart disease, diabetes, cancer etc. are not available consistently throughout the survey waves. However, using dummy variable adjustment for missing values, we estimate the model by including controls for several chronic conditions (such as diabetes, heart disease, hypertension, bronchitis, thrombosis, skin cancer and sexually transmitted diseases) and find that the results are robust. The results are available upon request.

^16^
The Anatomical Therapeutic Chemical (ATC) code is a seven‐digit alphanumeric code that categorizes medications based on their site of action, therapeutic and chemical features (The Pharmaceutical Benefits Scheme, 2016)

^17^
It should be also noted that K10 is not a diagnostic tool though there is substantial correlation between more severe scores and clinical diagnoses.

^18^
It is important to highlight that the findings are based on the healthcare costs covered only by the MBS and PBS. The estimated effect excludes the costs of specialized state‐based mental health services and other services such as those provided by non‐profit organizations due to unavailability of data.
