# Supplementary material for: Excess healthcare costs of psychological distress in young women: Evidence from linked national Medicare claims data
Source: Health Econ. 2022 Dec 10;32(3):715–34. doi: 10.1002/hec.4641 (PMC10947058; doi:10.1002/hec.4641)
Supplement: Supplementary file 1 — Supplementary Material [file HEC-32-715-s001.pdf]

Supplementary Materials

Table S1: Kessler Psychological Distress Scale (K10)

Please tick the answer that is correct for you:

	In the past four weeks:	None of the time	A little of the time	Some of the time	Most of the time	All of the time
1.	About how often did you feel tired out for no good reason?					
2.	About how often did you feel nervous?					
3.	About how often did you feel so nervous that nothing could calm you down?					
4.	About how often did you feel hopeless?					
5.	About how often did you feel restless or fidgety?					
6.	About how often did you feel so restless you could not sit still?					
7.	About how often did you feel depressed?					
8.	About how often did you feel that everything was an effort?					
9.	About how often did you feel so sad that nothing could cheer you up?					
10.	About how often did you feel worthless?					

Table S2: Summary statistics of covariates

	Full sample (N=53,449)		Level of psychological distress							
			No (N = 24,871)		Mild (N=11,008)		Moderate (N=7,745)		Severe (N=9,825)	
	Mean	SD	Mean	SD	Mean	SD	Mean	SD	Mean	SD
Age (min =17, max =28)	22.18	2.30	22.49	2.28	22.13	2.27	22.00	2.26	21.58	2.27
**Education**										
Grade 10 and below	0.02	0.15	0.01	0.12	0.02	0.14	0.02	0.15	0.05	0.21
Grade 11	0.03	0.16	0.02	0.13	0.02	0.15	0.03	0.16	0.05	0.22
Grade 12	0.31	0.46	0.28	0.45	0.32	0.46	0.33	0.47	0.35	0.48
Certificate	0.19	0.39	0.16	0.37	0.19	0.39	0.22	0.41	0.25	0.43
Diploma	0.08	0.27	0.07	0.25	0.08	0.27	0.08	0.27	0.09	0.28
Degree	0.31	0.46	0.37	0.48	0.31	0.46	0.26	0.44	0.18	0.38
Post graduate	0.07	0.25	0.09	0.29	0.06	0.24	0.06	0.23	0.04	0.19
**Marital Status**										
Never married	0.70	0.46	0.67	0.47	0.71	0.45	0.72	0.45	0.74	0.44
Married	0.07	0.25	0.08	0.28	0.06	0.23	0.05	0.22	0.04	0.20
De-facto	0.23	0.42	0.24	0.43	0.23	0.42	0.23	0.42	0.22	0.41
Seperated/divorced	0.00	0.06	0.00	0.04	0.00	0.06	0.01	0.07	0.01	0.08
**Income management**										
Impossible	0.03	0.18	0.02	0.12	0.02	0.15	0.03	0.18	0.08	0.27
Difficult	0.52	0.50	0.42	0.49	0.55	0.50	0.61	0.49	0.67	0.47
Easy	0.45	0.50	0.57	0.50	0.43	0.49	0.35	0.48	0.25	0.43
Access to healthcare card	0.28	0.45	0.23	0.42	0.28	0.45	0.32	0.47	0.39	0.49
**Employment status**										
Not unemployed	0.84	0.37	0.89	0.32	0.85	0.36	0.81	0.39	0.72	0.44
Unemployed	0.16	0.37	0.11	0.31	0.15	0.36	0.19	0.38	0.28	0.45
**Residence**										
Remote	0.01	0.11	0.01	0.12	0.01	0.11	0.01	0.10	0.01	0.09
Regional	0.23	0.42	0.24	0.42	0.23	0.42	0.23	0.42	0.24	0.43
Metro	0.75	0.43	0.74	0.44	0.76	0.43	0.76	0.43	0.75	0.43

Note. All variables except age are denoted as dummy variables.

Table S3: Coefficient estimates of the effects of psychological distress on various MBS items

	Imaging		Operations		Optometry		Other	
	(1)	(2)	(3)	(4)	(5)	(6)	(7)	(8)
	Positive costs	Log costs	Positive costs	Log costs	Positive costs	Log costs	Positive costs	Log costs
**Panel A**								
K10 score	0.0001	0.0004	0.0003	0.0010	0.0011**	-0.0002	0.0007**	0.0026
	(0.000)	(0.002)	(0.000)	(0.003)	(0.000)	(0.001)	(0.000)	(0.004)
Observations	53,449	20,560	53,449	6,498	53,449	17,813	53,449	7,425
**Panel B**								
Ref. group: No distress								
Mild	-0.0047	0.0034	-0.0042	-0.0039	0.0104	-0.0329**	0.0099*	-0.0175
	(0.007)	(0.026)	(0.005)	(0.053)	(0.007)	(0.016)	(0.005)	(0.066)
Moderate	0.0048	0.0215	0.0012	-0.0475	0.0063	0.0153	0.0118*	-0.0617
	(0.009)	(0.032)	(0.006)	(0.062)	(0.008)	(0.019)	(0.006)	(0.080)
Severe	-0.0003	-0.0260	0.0027	0.0029	0.0200**	-0.0154	0.0087	0.0234
	(0.010)	(0.035)	(0.007)	(0.071)	(0.009)	(0.021)	(0.007)	(0.084)
Observations	53,449	20,560	53,449	6,498	53,449	17,813	53,449	7,425

Note. Robust standard errors in parenthesis, clustered at individual level. *** p<0.01, ** p<0.05, * p<0.1. All regression models control for age, marital status, education, employment, income management, access to healthcare card, residence, state and year dummies and individual fixed-effects. Positive costs denote the extensive margin of healthcare costs, while log costs denote the intensive margin, conditional on incurring any healthcare costs.

Table S4: Coefficient estimates from monthly analysis

Part I

	Positive costs												
	(1) **T1**	(2) **T2**	(3) **T3**	(4) **T4**	(5) **T5**	(6) **T6**	(7) **T7**	(8) **T8**	(9) **T9**	(10) **T10**	(11) **T11**	(12) **T12**	(13) **Annual**
**Panel A**													
K10 score	0.0049***	0.0037***	0.0027***	0.0019***	0.0014***	0.0011***	0.0010***	0.0008***	0.0007***	0.0005***	0.0004**	0.0004**	0.0003**
	(0.000)	(0.000)	(0.000)	(0.000)	(0.000)	(0.000)	(0.000)	(0.000)	(0.000)	(0.000)	(0.000)	(0.000)	(0.000)
Observations	53,449	53,449	53,449	53,449	53,449	53,449	53,449	53,449	53,449	53,449	53,449	53,449	53,449
**Panel B**													
Ref group: No distress													
Mild	0.0312***	0.0229***	0.0234***	0.0169***	0.0136***	0.0097**	0.0089**	0.0086***	0.0061**	0.0041	0.0052**	0.0054**	0.0016
	(0.007)	(0.007)	(0.006)	(0.005)	(0.004)	(0.004)	(0.004)	(0.003)	(0.003)	(0.003)	(0.002)	(0.002)	(0.002)
Moderate	0.0545***	0.0319***	0.0304***	0.0222***	0.0160***	0.0116**	0.0121***	0.0114***	0.0094***	0.0077**	0.0072**	0.0071***	0.0066**
	(0.009)	(0.008)	(0.007)	(0.006)	(0.005)	(0.005)	(0.004)	(0.004)	(0.003)	(0.003)	(0.003)	(0.003)	(0.003)
Severe	0.0872***	0.0565***	0.0434***	0.0345***	0.0231***	0.0159***	0.0146***	0.0094**	0.0089**	0.0081**	0.0067*	0.0058*	0.0049
	(0.010)	(0.009)	(0.007)	(0.006)	(0.006)	(0.005)	(0.005)	(0.004)	(0.004)	(0.004)	(0.003)	(0.003)	(0.003)
Observations	53,449	53,449	53,449	53,449	53,449	53,449	53,449	53,449	53,449	53,449	53,449	53,449	53,449

Part II

	Log costs												
	(1) **T1**	(2) **T2**	(3) **T3**	(4) **T4**	(5) **T5**	(6) **T6**	(7) **T7**	(8) **T8**	(9) **T9**	(10) **T10**	(11) **T11**	(12) **T12**	(13) **Annual**
**Panel C**													
K10 score	0.0170***	0.0189***	0.0203***	0.0196***	0.0195***	0.0182***	0.0173***	0.0168***	0.0167***	0.0163***	0.0156***	0.0147***	0.0157***
	(0.002)	(0.001)	(0.001)	(0.001)	(0.001)	(0.001)	(0.001)	(0.001)	(0.001)	(0.001)	(0.001)	(0.001)	(0.001)
Observations	27,653	37,322	42,590	45,553	47,385	48,650	49,592	50,281	50,806	51,221	51,525	51,740	51,957
**Panel D**													
Ref group: No distress													
Mild	0.0494**	0.0949***	0.0925***	0.1025***	0.1036***	0.0931***	0.0899***	0.0796***	0.0879***	0.0875***	0.0805***	0.0736***	0.0796***
	(0.025)	(0.020)	(0.018)	(0.017)	(0.016)	(0.015)	(0.015)	(0.014)	(0.014)	(0.013)	(0.013)	(0.013)	(0.013)
Moderate	0.1874***	0.2093***	0.1993***	0.1937***	0.2049***	0.1879***	0.1758***	0.1646***	0.1698***	0.1675***	0.1537***	0.1418***	0.1522***
	(0.029)	(0.024)	(0.022)	(0.020)	(0.019)	(0.018)	(0.018)	(0.017)	(0.017)	(0.016)	(0.016)	(0.015)	(0.016)
Severe	0.2815***	0.3423***	0.3551***	0.3362***	0.3292***	0.3085***	0.2981***	0.2881***	0.2794***	0.2738***	0.2617***	0.2457***	0.2637***
	(0.032)	(0.027)	(0.025)	(0.023)	(0.022)	(0.021)	(0.020)	(0.019)	(0.019)	(0.018)	(0.018)	(0.017)	(0.018)
Observations	27,653	37,322	42,590	45,553	47,385	48,650	49,592	50,281	50,806	51,221	51,525	51,740	51,957

Note. Robust standard errors in parenthesis, clustered at individual level. *** p<0.01, ** p<0.05, * p<0.1. All regression models control for age, marital status, education, employment, income management, access to healthcare card, residence, state and year dummies and individual fixed-effects. Positive costs denote the extensive margin of healthcare costs, while log costs denote the intensive margin, conditional on incurring any healthcare costs.

Table S5: Conditional and unconditional marginal/incremental effects of psychological distress on out-of-pocket estimates (dollars)

	Total Healthcare Cost		MBS		PBS	
	Conditional OOP costs ($)	Unconditional OOP costs ($)	Conditional OOP costs ($)	Unconditional OOP costs ($)	Conditional OOP costs ($)	Unconditional OOP costs ($)
**Panel A**						
K10 score	3.90	3.48	4.43	2.60	1.06	0.87
**Panel B**						
Psychological distress	31.94	28.99	40.54	24.48	8.55	7.17

	Specialist Items Consultant Psychiatrist		Allied Health Clinical psychologist, psychologist, occupational therapist and social worker	
	Conditional OOP costs ($)	Unconditional OOP costs ($)	Conditional OOP costs ($)	Unconditional OOP costs ($)
**Panel A**				
K10 score	9.69	0.26	6.83	0.58
**Panel B**				
Psychological distress	147.86	5.66	68.61	9.58

Note. The conditional OOP costs are the marginal/incremental effects in dollar scale estimated from the second part of the model. The unconditional predicted OOP costs are derived manually by multiplying the predicted probabilities and OOP costs from each part of the two-part model. Duan smearing factor by K10 scores is used to transform predictions of log expenditures into its levels. All costs are in 2019 Australian dollars (AUD) and adjusted for inflation.

Table S6: Coefficient estimates from balanced panel

	(1) Positive costs	(2) Log costs
**Panel A**		
K10 score	0.0006***	0.0174***
	(0.000)	(0.001)
Observations	26,215	25,483
**Panel B**		
Psychological distress (=1 if K10 >24)	0.0076***	0.1690***
	(0.003)	(0.017)
Observations	26,215	25,483
**Panel C**		
Reference group: No distress	0.0012	0.0777***
Mild	(0.003)	(0.017)
	0.0092***	0.1604***
Moderate	(0.003)	(0.021)
	0.0066	0.2913***
Severe	(0.004)	(0.024)
Observations	26,215	25,483

Note. Robust standard errors in parenthesis, clustered at individual level. *** p<0.01, ** p<0.05, * p<0.1. All regression models control for age, marital status, education, employment, income management, access to healthcare card, residence, state and year dummies and individual fixed-effects. Positive costs denote the extensive margin of healthcare costs, while log costs denote the intensive margin, conditional on incurring any healthcare costs.

Table S7: Coefficient estimates from alternate measures of depression

	K10 Score		Diagnosed/treated for depression in the past year		Felt depressed in the past year	
	(1)	(2)	(3)	(4)	(5)	(6)
	Positive cost	Log Cost	Positive cost	Log Cost	Positive cost	Log Cost
**Panel A**						
Psychological distress	0.0050**	0.1475***	0.0049	0.1900***	0.0066***	0.1649***
	(0.002)	(0.014)	(0.004)	(0.021)	(0.002)	(0.014)
Observations	44,790	43,557	44,781	43,548	44,785	43,552
**Panel B**						
Controlling for physical health status and conditions						
Psychological distress	0.0044*	0.1353***	0.0041	0.1832***	0.0060**	0.1574***
	(0.002)	(0.014)	(0.004)	(0.021)	(0.003)	(0.014)
Observations	44,526	43,296	44,517	43,287	44,521	43,291

Note. Robust standard errors in parenthesis, clustered at individual level. *** p<0.01, ** p<0.05, * p<0.1. All regression models control for age, marital status, education, employment, income management, access to healthcare card, residence, state and year dummies and individual fixed-effects. Positive costs denote the extensive margin of healthcare costs, while log costs denote the intensive margin, conditional on incurring any healthcare costs.

Table S8: Utilisation of mental health services

	Level of psychological distress			
	No	Mild	Moderate	Severe
**GP**				
GP attendances (23,36)	80.0	77.9	75.2	71.0
Mental health - GP (credentialed with CEM) (2700-2725)	2.5	4.8	7.3	10.5
Other	17.5	17.3	17.5	18.5
**Specialist**				
Specialist or consultant physician (104, 105, 110, 116)	78.9	65.7	56.1	35.8
Consultant psychiatrist (301, 304, 306, 319)	10.5	21.5	28.8	49.2
Other	10.6	12.7	15.1	15.0
**Allied**				
Clinical Psychologist (80010)	38.8	43.6	44.6	42.1
Psychologist (80110)	36.5	39.2	38.7	41.6
Occupational therapist and Social Worker (80135, 80160)	4.0	3.4	4.0	3.9
Other	20.7	13.8	12.7	12.5
**PBS use**				
NO5A - Antipsychotics	0.5	0.8	1.3	3.0
NO5B - Anxiolytics	0.5	0.9	1.2	2.2
NO5C - Hypnotics and sedatives	0.4	0.5	0.6	0.9
NO6A - Antidepressants	12.8	23.0	29.8	37.2
NO6B - Psychostimulants and nootropics	0.6	1.0	1.0	0.9
Total use mental health related ATC codes	14.8	26.1	33.9	44.1
